# Report on Dysentery, Made to the Cook Co. Medical Society

**Published:** 1852-11

**Authors:** A. B. Palmer


ARTICLE II.—*Report on Dysentery, made to the* COOK CO. MEDICAL SOCIETY, by A. B. PALMER, M. D.

[This article was prepared for the editorial department of the last No. of the Journal, but was unavoidably crowded out. The Report was a verbal one, and this abstract has been made up from notes taken at the time.—*Eds. Jour.*]

THE subject of Dysentery was taken up for the evening's consideration. Dr. Palmer commenced by defining the disease—an inflammation of the mucous membrane of the large intestines—but considered it, at least in many cases, especially when occurring as an epidemic, or endemic, as an inflammation of a peculiar character, differing from the inflammation that occurs in accidental Enteritis, besides in location. He thought a simple inflammation of the mucous membrane of the large intestines, produced by a local or accidental cause, would not give rise to all the phenomena which usually attend a case of genuine Dysentery.

Writers divide Acute Dysentery into *Bilious*—where there is special derangement of the hepatic secretion—whether that consists of deficiency or superabundance of bile; *Adynamic*—where there is special depression of the vital forces; *Intermittent* and *Remittent*—where the disease is connected with distinct and well developed paroxysmal or miasmatic fever, or in the absence of such fever, the dysenteric symptoms have regular periods of decided exacerbation—Typhus Dysentery complicated with Typhoid fevers—Rheumatic Dysentery, he said, was also mentioned by authors, occurring as the result of a metastasis of rheumatic inflammation from some other part, but of this form of the disease he had no personal knowledge. He doubted whether rheumatic inflammation affected in that way a mucous membrane.

He remarked that Dysentery was sometimes complicated with *general Enteritis.* In these cases there was not unfrequently, in addition to the bloody mucus, and discharges of moderate quantities of fresh blood, free sero-sanguinous discharges of a briny or much darker appearance, the color of the blood being darkened by the action of the secretions of the small intestines. Other symptoms also marked this complication.

Cholera was sometimes complicated with Dysentery. He had seen many cases of this kind during the present season. Also cholera complicated with general Enteritis and Dysentery. In cases of this kind the patients were attacked with cholera in the usual way. Diarrhœa becoming serous, attended with vomiting, cramps &c., but with less tendency to the blue stage, and the profuse, cold perspiration; reaction occurring earlier, tenderness, tenesmus, fever and bloody discharges after 24 to 72 hours. When the small intestines are also involved, in addition to the dysenteric discharges, more free sero-sanguinous evacuations occur. These cases, according to his observation, were less liable to prove speedily fatal, than where a tendency to the complication did not exist, in consequence of the early disposition to reaction; and the inflammatory symptoms he had been very generally able to subdue. He often prognosticated on first seeing patients with active rice-water discharges, severe cramps, &c., that Dysentery or Enteritis would occur, from a greater rapidity of pulse, more tendency to dryness of the tongue, slight tenderness (in some cases) of the abdomen, more frequent but less profuse discharges, a degree of pain aside from the cramp, and above all, from a degree of warmth not present with the same amount of rice-water discharges where this tendency does not exist. In these cases, he thought the violent determination of blood to the internal organs, and especially to the mucous membrane of the alimentary canal, occurring in cholera, produced active congestion, and developed inflammation.

The *prognosis* in simple sporadic dysentery, was usually favorable, but in epidemic and endemic it was often exceedingly unfavorable. He has seen at least one endemic prevalence of it that was more fatal than almost any other acute disease he knew.

Anatomical lesions in Dysentery, consisted in congestion, inflammation, and its products of softening, abrasion, ulceration, and gangrene of the mucous membrane of the large intestines.

The causes of the disease were various—as congestion of the liver and obstruction of circulation through it, the effect of cold and moisture, determining the blood internally, accumulations of fæces, or the existence of other irritating secretions or substances in the intestines, the disturbance of the circulation, internal congestion produced by a paroxysm of miasmatic fever, particularly the cold stage; and, as already stated, by the determination of blood to the mucous membrane, in cholera, &c.: but where it prevails as an epidemic or endemic, it appears to depend upon a specific cause involved in as much obscurity as the cause of cholera.—Some have regarded contagion as a cause, but he thought there was no evidence to prove that there was a poison found in the bodies of persons sick with dysentery, which, when applied to other persons, produced the disease in them—and this circumstance was necessary, in his view of the subject, to make it contagious. Such a poison was formed in small pox, measles, and all other diseases known to be contagious, and was the essential fact of contagion.—If this simple view of contagion was universally taken by the profession, as it should be, there would be a clearer understanding of, and less disputation about the matter.

The treatment of dysentery must be varied according to the type of the disease, and the causes which produce it.

Bleeding, in many sporadic cases of dysentery, and in some epidemics—indeed in all cases of a high sthenic type, is often very beneficial, and cannot safely be dispensed with. It should be practiced early, and by arresting the course of the disease, will often prevent that debility which is such a source of apprehension to many practitioners. Local bleeding, as by cups to the abdomen and leeches to the arms, was also useful. Of course, these remedies are not applicable in adynamic cases.

Emetics are recommended, but he would give them only when there was evidence of matters in the stomach causing irritation there, and which would be likely to irritate the inflamed surfaces, in passing over them in seeking an outlet in the natural way.

He gives *cathartics* only when necessary to carry off irritating matters from the bowels. He has seen more of Scybala in the books than in practice. They sometimes exist, but not as frequently as many writers would leave us to suppose.

The mildest cathartics should be used. Castor oil, or emulsions of oil with a moderate quantity of turpentine, or next, the neutral salts.

Diaphoretics are often useful adjuvants to more decided treatment, and ipecac may be given when the stomach is not irritable, throughout the earlier stages of the disease.

Tart. antimony was strongly recommended by Dr. S. Jackson, formerly of Northumberland, now of Philadelphia, Pennsylvania. Dr. J. regards the disease as an inflammation, and uses the Ant. as an antiphlogistic agent. He believes it does not directly reach the seat of the disease, to produce a local irritating effect. Dr. J.'s opinions are regarded as valuable by those who know him, but he (Dr. P.) has not used the remedy in that way.

Opium, Dr. P. regards as an indispensable agent in the correct treatment of dysentery, and uses it from the beginning to the end of the disease—much of the time in connection with other means. But he depends upon it to allay pain, procure rest, cause diaphoresis, and by a direct action which opium is capable of, to subdue the inflammation. He gives from two to four grains sufficiently often to keep up a decided anodyne influence. He has not ventured upon the poisonous doses of from ten to twenty grains, as advised by some in abdominal inflammations.

Mercury is recommended by some writers to be carried to the extent of salivation, to overcome the inflammation. Dr. P. regards the indiscriminate or general use of the article to that extent as a great evil, but in many cases, indeed in a majority of cases, and especially where there is evidence of biliary obstruction, as there very often is, he would commence the treatment with two or three 5-grain doses joined with opium, and after twenty-four or forty-eight hours followed by castor oil, &c. He is very confident, whatever may be the speculations of chemists, or physiologists, that mercury operates most decidedly and beneficially—much more efficiently than alkalies or any other agent in removing engorgement of the liver, and exciting the biliary secretion. It is an article very much abused, doubtless, but which in the present state of our knowledge cannot be entirely dispensed with in this, as in many other diseases, without a loss to suffering humanity. In the later stages of the disease, he had often seen a few 2-grain doses followed by the most decided relief.

Acetate of lead: Dr. P. has not a very favorable opinion of this remedy, in dysentery, when given by the stomach. It often disturbs that organ when given in sufficient doses and for a sufficient length of time to produce much impression upon the disease. It is indicated with opium in hæmorrhage of the small intestines.

He has not used Sulph. Zinc. or copperas in this disease. Nit. of silver may do much good in the latter stages, and particularly if ulcerations exist, and should be given by enema.

Nitric acid. He has not used this much—has seen it given, but has not been favorably impressed with its effects—has seen rapid improvement where it has been discontinued and opiates used.

Oil of turpentine and Bals. of Copaiba. He has a very favorable opinion of turpentine in dysentery, and indeed in many cases of inflammation, where it can be brought in direct contact with the inflamed part, it modifies the condition of the part, and overcomes the inflammation. He has found turpentine very beneficial in sub-acute stages of the disease. His usual formula is

Oil Terebinth. ʒiij.<br>

Tinct. Opii from ʒjss to ʒiij.<br>

Gum Acacia }<br>

Sugar } aa. ℥ss.<br>

Aqua Camphora ℥ij. M.

A large teaspoonful once in from two to four hours.

In the ordinary cases of dysentery occurring this season (they have not generally been severe,) not accompanied with an intermitting tendency, the following treatment has with him almost universally been successful.

For the first twenty-four or thirty-six hours, two to four grains of opium once in three hours: to which has been added generally from six to twelve grains of sub-muriate Hydrarg. in from three to six grain doses, in two or three of the first doses of opium. This, followed by a cathartic dose of castor oil, and oil of turpentine, when no diarrhœa had preceded or accompanied the disease

This followed by the turpentine emulsion, with the addition of as much opium or morphine as was necessary to control the discharges. If much blood was discharged, or if this course was not readily successful, a few enemas of acetate of lead and laudanum would complete the cure.

When an intermitting tendency existed, whether manifested by that character of the dysenteric symptoms, or by distinct febrile paroxysms, Quinine was the great remedy. A scruple to ʒss, combined with a grain or more of morphine, given in four doses, during one intermission or remission, seldom, almost never, failed to remove both the fever and the dysentery. Indeed, quinine often acts well, when scarcely any intermitting tendency can be traced. He thinks quinine one of the few really curative agents. Many other remedies seem to palliate, but quinine, when it is applicable, *cures.*

Nux Vomica. Knows little personally of the effects of this in dysentery. Has seen it used, but not sufficiently to form an opinion from observation of its value.

A diet producing the smallest amount of residuum, and bland mucilaginous drinks should not be neglected.

Topical remedies. Cold water, or water at least several degrees below the temperature of the body, applied externally, and by enema—often useful, on the principle that cold reduces inflammation. It must not be used so cold as to produce a shock, and must be repeated sufficiently often to continue the effect. Where cold does not agree, as it sometimes will not, fomentations and emolient enemas will often afford relief.

Rubefacients and blisters often *very* beneficial in the latter stages.

Enemas are *necessary* in the proper treatment of severe cases of dysentery. These may be emolient, anodyne, astringent, or caustic, as indicated by the condition. There are few medical diseases where remedies can be applied directly to the diseased part. This is one, and the opportunity should not be neglected. Where the bloody discharge is free and exhausting, injections of acetate of lead, and laudanum, in cold water, are indispensable, and enemas of Nit. silver, in the last stages, when ulcerations exist, are attended with the greatest benefit. In some cases, especially in children, and where they are most needed, in consequence of constant tormina, anodyne enemas, however small the quantity, are not retained, and here suppositories of opii, or where they are rejected, an Oint. of morphine and lard applied about and within the sphincter with the finger, is often of signal service.

